# Acute live firefighting effects on ventricular‐arterial coupling and pulsatile afterload in middle‐aged firefighters

**DOI:** 10.14814/phy2.70659

**Published:** 2025-11-14

**Authors:** João L. Marôco, Abbi D. Lane, Sushant M. Ranadive, Huimin Yan, Kanokwan Bunsawat, Gavin P. Horn, Denise L. Smith, Tracy Baynard, Bo Fernhall

**Affiliations:** ^1^ Integrative Human Physiology Laboratory, Manning College of Nursing & Health Sciences University of Massachusetts Boston Boston Massachusetts USA; ^2^ Department of Exercise and Health Sciences University of Massachusetts Boston Boston Massachusetts USA; ^3^ School of Kinesiology University of Michigan Ann Arbor Michigan USA; ^4^ Department of Kinesiology, School of Public Health University of Maryland College Park Maryland USA; ^5^ Division of Geriatrics, Department of Internal Medicine University of Utah Salt Lake City Utah USA; ^6^ Geriatric Research, Education, and Clinical Center, George E. Wahlen Department of Veterans Affairs Medical Center Salt Lake City Utah USA; ^7^ Department of Nutrition and Integrative Physiology University of Utah Salt Lake City Utah USA; ^8^ Department of Health, Sport, and Human Physiology University of Iowa Iowa City Iowa USA; ^9^ Fire Safety Research Institute, UL Research Institutes Columbia Maryland USA; ^10^ Illinois Fire Service Institute, University of Illinois‐Urbana/Champaign Champaign Illinois USA; ^11^ Department of Health and Human Physiological Sciences Skidmore College Saratoga Springs New York USA

**Keywords:** arterial elastance, heart vascular interactions, ventricular elastance

## Abstract

Firefighting increases afterload, leading to ventricular‐arterial coupling mismatch in young firefighters that may contribute to coronary hypoperfusion and the elevated risk of on‐duty cardiac events. Since this risk is higher with aging in firefighters, we examined their ventricular‐vascular coupling and afterload responses to acute firefighting. Twenty‐two male firefighters (40–59 years) performed 18‐min high‐intensity firefighting drills while wearing protective gear and breathing apparatus. Echocardiography was conducted before and within 10 min after firefighting to estimate cardiac volumes, while tonometry‐derived pulse wave analysis estimated wasted pressure effort (Ew) and aortic reservoir function. Ventricular‐arterial coupling was quantified using the arterial (Ea) to ventricular (Ees) elastance ratio, and coronary perfusion was estimated via the Buckberg index. Firefighting reduced stroke volume (difference (∆) = −17 mL, *p* < 0.001), Ew (∆ = −800 dyne cm^−2^ s, *p* = 0.005), aortic reservoir function (∆ = −6.9%, *p* < 0.001), and Buckberg index (∆ = −0.28, *p* < 0.001). Firefighting augmented Ea/Ees (∆ = 0.10, *p* = 0.035) stemming from increases in Ea (∆ = 0.16 mmHg.mL^−1^, *p* = 0.046) not counteracted by Ees. Heart rate changes were associated with Ew (*r* = −0.60, *p* = 0.017) and aortic reservoir function (*r* = −0.80, *p* < 0.001). Although middle‐aged firefighters exhibited typical post‐firefighting cardiovascular strain, including reduced coronary perfusion, the role of ventricular‐arterial interactions and pulsatile afterload remains unclear due to heart rate confounding.

## 1 INTRODUCTION

Firefighters face a disproportionately higher risk of on‐duty cardiovascular‐related death compared to other occupations, and this risk is expected to worsen with aging. The National Fire Protection reports that ~25% of firefighters are aged ≥50 and have a >20‐fold higher risk of sudden cardiac death during or shortly after fire suppression than young colleagues (20–39 years) (Fahy et al., 2022; Kales et al., 2007). Firefighting strains the cardiovascular system due to strenuous physical work, hyperthermia, pollutants, and psychological distress, with each factor alone or collectively contributing to higher on‐duty fatality rates among firefighters (Barr et al., 2010; Kales & Smith, 2017; Smith et al., 2013). Such risk is heightened by the unfavorable cardiometabolic risk profiles seen in older firefighters, specifically higher prevalences of obesity and hypertension (Moffatt et al., 2021; Smith et al., 2020). Thus, expanding knowledge of the cardiovascular response to fire suppression within aging contexts is crucial for prevention efforts.

The cardiovascular response to firefighting is well described in younger firefighters, comprising marked increases in both heart rate and blood pressure, along with reductions in plasma and stroke volume (SV) (Kales & Smith, 2017; Smith et al., 2001). Augmented cardiovascular strain is apparent immediately after fire suppression, as elevated HR and reduced SV persist (Fernhall et al., 2012). Depressed SV after firefighting stems from a dehydration‐induced fall in cardiac preload, but it also depends on afterload, because of a coupling mismatch between the left ventricle and arterial system (Hibner et al., 2022). Using the ventricular‐arterial coupling framework, we demonstrated that an increase in afterload (arterial elastance, Ea) not compensated by ventricular contractility (ventricular elastance, Ees) led to post‐firefighting reductions in SV (Hibner et al., 2022). The resultant increase in the coupling ratio (i.e., Ea/Ees) implies that the left ventricle did not increase contractility to overcome the increased afterload, suggesting a state of cardiovascular inefficiency and altered systolic and diastolic function (Fernhall et al., 2012; Hibner et al., 2022).

Although the ventricular‐arterial coupling concept offers valuable insights into heart‐vessel interactions, it overlooks two crucial physiological phenomena affecting pulsatile afterload: wave reflection and the Windkessel effect (Chirinos, 2013). Early reflections of the incident pressure waveform at bifurcations increase the work of the left ventricle, a phenomenon partly attributed to arterial stiffening, which is inconsistently observed in firefighting (Fahs et al., 2011; Hibner et al., 2022; Lane‐Cordova et al., 2015). Because the extra cardiac work to overcome the early reflection‐induced augmentation of aortic pressure fails to contribute to systolic ejection, it represents a wasted pressure effort (Ew) that may lead to post‐firefighting reductions in SV (Hashimoto et al., 2008). Firefighting might also reduce aortic compliance, providing a Windkessel‐type dampening of pulsatile flow and blood distribution during diastole (i.e., reservoir function) (Belz, 1995). Both early wave reflection and a reduced Windkessel effect contribute to coronary hypoperfusion reported after firefighting (Belz, 1995; Horn et al., 2011), possibly underlying the increased risk of on‐duty cardiac events. Based on these considerations, pulsatile afterload cannot be described by a single parameter, as it reflects the interplay of complex physiological phenomena. Nonetheless, pulsatile afterload is a plausible contributor to the cardiovascular strain of firefighting, particularly among aging firefighters, driven by aortic stiffening and hypertension (AlGhatrif et al., 2013; Mitchell et al., 2007; Stock et al., 2023).

Therefore, we investigated the ventricular‐arterial coupling and pulsatile afterload (i.e., Ew and aortic reservoir function) responses after live firefighting among middle‐aged firefighters. We hypothesized that: (1) the ventricular‐vascular coupling ratio would increase due to a rise in Ea not matched by an increase in Ees; and (2) Ew would increase, while aortic reservoir function would decline post‐firefighting.

## 2 METHODS

### 2.1 Experimental design

This study is a retrospective analysis of a placebo‐controlled, crossover trial examining the effects of aspirin supplementation on hemodynamic responses and inflammation to live firefighting (ClinicalTrials.gov, NCT01276691). The University of Illinois at Urbana‐Champaign Institutional Review Board approved the trial, and all testing procedures were aligned with the Declaration of Helsinki. The current analysis used data only from the placebo visit, given that no effect of aspirin was observed in resistive (e.g., mean arterial pressure) and pulsatile afterload parameters (e.g., central pulse wave velocity) after firefighting drills (Lane‐Cordova et al., 2015). Echocardiography and applanation tonometry measurements were conducted before and within 10‐min post‐firefighting drills in lateral and dorsal decubitus positions, respectively. All participants were tested in the mornings under an euhydrated state and consumed standardized meals as detailed previously in a dimmed light and climate‐controlled laboratory room (~22°C) (Smith et al., 2019).

### 2.2 Participants

Twenty‐two middle‐aged male and female firefighters (range: 40–59 years) were recruited from fire departments in Illinois, USA, and via the Fire Service Association, but only male firefighters volunteered for the study. Exclusion criteria included: coronary artery and renal disease, contraindications to aspirin, current or recent use of NSAIDs, clopidogrel, warfarin, or physician‐prescribed aspirin. All firefighters underwent a medical evaluation based on NFPA 1582 guidelines, which included cardiovascular risk assessment (lipid profiles) and a treadmill stress test to 85% of age‐predicted maximum heart rate (HR) to estimate maximal oxygen consumption. All firefighters provided written informed consent.

### 2.3 Firefighting drills

All participants completed 18 min of standardized high‐intensity, simulated live‐fire activities, while wearing full personal protective equipment and a self‐contained breathing apparatus (SCBA, ∼20 kg). All drills followed a 2‐min work and 2‐min rest cycle and were conducted on the second floor of a concrete and steel two‐story structure containing live fires. Drills included stair climbing, simulated forcible entry, and hose advancement; the complete characterization is described elsewhere (Lane‐Cordova et al., 2015). HR was monitored using a Polar watch (Polar Electro Inc., Bethpage, NY). Upon drill completion, participants were transported to the laboratory by a golf cart. Structural temperatures were controlled (floor~35–41°C, and above‐floor ~70–82°C) (Lane‐Cordova et al., 2015).

### 2.4 Echocardiography

Cardiac function was estimated using 2D echocardiography (Aloka Alpha‐10, Tokyo, Japan). Using M‐mode, left ventricular dimensions in systole and diastole were averaged from three measurements in the parasternal long‐axis view. The end‐diastolic diameter was measured distally to the mitral valve leaflets, from the edge of the septum to the endocardial surface of the posterior wall at R‐wave onset. The end‐systolic diameter was considered the shortest distance between the trailing edge of the septum and the posterior wall. Left‐ventricular volumes were estimated using the Teichholz Equation (Pombo et al., 1971). SV was calculated as the end‐diastolic volume (EDV) minus end‐systolic volume (ESV). Cardiac output (CO) was calculated as the product of HR and SV. Ejection fraction, a systolic function index, was determined using this equation: [(SV/EDV) × 100]. Peak mitral inflow velocities (E, A, E/A), parameters of diastolic function, were measured using pulsed Doppler echocardiography from the apical four‐chamber view with a 2.5–5.0 MHz transducer, averaging the three to five highest values. Longitudinal myocardial velocities at the lateral mitral valve ring in the apical four‐chamber were assessed using tissue Doppler imaging with peak e' and peak‐S taken as parameters of diastolic and systolic function, respectively. The peak S′ /e' ratio was taken as an index of systolic‐diastolic coupling, where higher or increased ratios reflect reduced coupling (Friedberg et al., 2016). All cardiac measurements were recorded during spontaneous breathing. Diastolic dysfunction was determined when two of the following criteria were observed: peak septal e′ < 7 cm/s; peak lateral e′ < 10 cm/s, and E/e′ ratio > 14, while systolic dysfunction was defined as EF < 50% (Lang et al., 2015; Nagueh et al., 2016).

### 2.5 Brachial blood pressure

Brachial systolic (bSBP) and diastolic blood pressure (bDBP) were measured twice, 1 min apart, using an oscillometric cuff (HEM‐907 XL; Omron, Japan) at heart level after a 10‐min supine rest in a quiet room. When readings differed by >5 mmHg, additional measurements were taken until two consecutive values were <5 mmHg. Mean arterial pressure (MAP) was calculated as (SBP + 2 × DBP)/3. Hypertension was defined as seated SBP ≥130 mmHg and/or DBP ≥80 mmHg, or based on the use of antihypertensive medication (Whelton et al., 2018).

### 2.6 Pulse wave contour analyses

Applanation tonometry measurements of the radial artery were conducted in duplicate before and after the firefighting drills with participants in the supine position over a 10‐s epoch (Millar Instruments, Houston, TX). Radial waveforms were calibrated using bSBP and bDBP. A validated transfer function (SphygmoCor; AtCorMedical, Sydney, Australia) was applied to reconstruct the aortic pressure waveform, estimating central BP, end‐systolic pressure (ESP), and augmentation index (AIx) (Sharman et al., 2006). AIx was calculated as the ratio of augmentation pressure (difference between the first and second systolic shoulders) to pulse pressure. Since AIx is HR‐dependent, it was normalized to 75 bpm (AIx75) (Wilkinson et al., 2000). Measurements required in‐device quality scores of >80%.

Central pulse wave velocity (cPWV) was also assessed, wherein common carotid and femoral pressure waveforms were recorded using a high‐fidelity strain‐gauge transducer. Transit times were estimated from the ECG R‐wave to the foot of the waves using the intersecting tangent foot‐to‐foot method. cPWV was calculated by dividing the measured carotid‐femoral distance by the transit time (Townsend et al., 2015). Coefficients of variation for cPWV and AIx in our laboratory are <5%.

The Buckberg index, an indirect estimate of myocardial perfusion, was calculated as the diastolic pressure time index (DPTI = DBP × diastolic time) divided by the systolic pressure time index (SPTI = SBP × ejection time).

### 2.7 Ventricular‐arterial coupling and pulsatile afterload analyses

#### 2.7.1 Ventricular arterial coupling

Ea was calculated as ESP/SV, and Ees as ESP/ESV (Chantler et al., 2008; Sunagawa et al., 1983). ESP was derived from aortic pressure waveforms (Kappus et al., 2013). For the Ees estimation, the pressure‐volume loop intercept (V_0_) was assumed to be zero, as its actual value is negligible and cannot be determined noninvasively (Sunagawa et al., 1983).

#### 2.7.2 Pulsatile afterload parameters

##### Wasted pressure effort

Ew was calculated using the equation Ew = 2.09 × AP×(ED − Tr), where AP (augmented pressure) is the difference between systolic pressure and the peak pressure from initial pressure and wave reflection (Hashimoto et al., 2008). ED represents the total ejection duration, while Tr is the systolic travel time of the propagated wave. The constant 2.09 approximates half the area of an ellipse, reflecting the shape of the wasted pressure portion in the reconstructed aortic waveform (Hashimoto et al., 2008). Ew derived from pressure‐only analysis can lead to physiologically implausible negative values (i.e., the inflection point occurs after the systolic peak). To address this pitfall, previously discussed by our group, Ew is also reported using only positive values (Ew >0) (Marôco et al., 2024).

##### Pulsatile afterload—Aortic reservoir function

As advanced by Levenson et al. (1981), the aortic reservoir function is expressed as the ratio of diastolic run‐off (i.e., blood volume distributed from the aorta) to stroke volume. The diastolic run‐off was estimated as: systemic arterial compliance × ESP − diastolic pressure. Systemic arterial compliance was estimated as the ratio of tau (τ) to total peripheral resistance (TPR). τ was defined as: diastolic time/ln (ESP−DBP), while TPR was calculated as: MAP/CO (obtained from echocardiography).

### 2.8 Statistical analyses

All statistical analyses were performed using R software (version 4.4.0) with a significance level set at *α* = 0.05. Data are reported as mean (SD) unless otherwise specified. The normality of characteristics and primary outcomes was assessed using the Shapiro–Wilk test and inspection of QQ plots.

Paired *t*‐tests were used to examine the pre‐to‐post firefighting changes in cardiac function, ventricular vascular coupling, and pulsatile afterload parameters. Hedge's effect size was computed for comparisons and interpreted following Cohen's benchmarks: small (*g* < 0.2), medium (*g*: 0.2–0.5), and large (*g*: 0.5–0.8). Pearson correlation coefficients were used to test associations between firefighting‐induced changes in cardiac function, ventricular‐arterial coupling, pulsatile afterload, and coronary perfusion parameters. To control for the effects of HR, obesity, and hypertension on the main outcomes in response to firefighting activities, ANCOVA models were used.

## 3 RESULTS

### 3.1 Characteristics of participants

The clinical characteristics of the firefighters are depicted in Table 1. Thirty‐five percent of the firefighters had hypertension (*n* = 7), while 48% had obesity and high LDL (*n* = 11). All firefighters met diastolic dysfunction criteria based on peak lateral e′ < 10 cm/s and peak septal e′ < 7 cm/s, while 10 firefighters also met the criterion of E/e′ > 14. Systolic function was normal, as indexed by EF.

**TABLE 1:** Characteristics of the participants (*n* = 22).

Age, years	48 (6)
Body mass index, kg/m^2^	29.88 (6.08)
Waist circumference, cm	94 (24)
LDL, mg/dL	129.43 (34.21)
HDL, mg/dL	47.33 (11.88)
Total cholesterol, mg/dL	200.68 (35.94)
Age‐predicted HR_max_, bpm	160 (9)
Predicted VO_2 max_, ml/kg/min	41.13 (9.58)

*Note*: Data presented as mean (SD).

Abbreviations: bDBP, brachial diastolic blood pressure; bSBP, brachial systolic blood pressure; cIMT, carotid intima media thickness; HDL, high‐density lipoprotein; HR, heart rate; LDL, low‐density lipoprotein; VO_2 max_, maximal oxygen uptake.

### 3.2 Post‐firefighting responses

#### 3.2.1 Cardiac function

Firefighting reduced EDV (delta (∆) = −18 mL, 95% CI: −31 to −4 mL, *t* (21) = −2.75, *p* < 0.001) and consequently SV (∆ = −17 mL, 95% CI: −26 to −8 mL, *p* < 0.001) and systolic function as indexed by EF (Table 2). Post‐firefighting reductions were also observed in diastolic function as indexed by peak mitral E wave (∆ = −12 cm/s, 95% CI: −17 to −6 mL, *p* < 0.001) and peak e′ lateral (∆ = −9 cm/s, 95% CI: −12 to −7 mL, *p* < 0.001). The lateral (∆ = 0.82, 95% CI: 0.13–1.51, *p* = 0.024) but not septal S′/e′ was increased after firefighting.

**TABLE 2:** Cardiac function responses to acute live‐firefighting.

	*n*	Pre	Post	*p* (g)
Systolic function				
EDV, mL	22	135 (23)	119 (34)	0.012 (−0.55)
ESV, mL	22	46 (13)	46 (19)	0.888 (0.00)
SV, mL	22	89 (18)	73 (18)	<0.001 (−0.88)
EF, %	22	66 (7)	63 (6)	0.033 (−0.51)
Peak S′ (lat), cm/s	18	13.4 (4.2)	13.3 (3.9)	0.419 (−0.03)
Peak S′ (sep), cm/s	21	15.0 (4.4)	13.0 (3.1)	0.120 (−0.51)
Diastolic function				
Peak E‐wave, cm/s	22	71.0 (16.0)	60.2 (15.5)	<0.001 (−0.67)
Peak A‐wave, cm/s	22	61.2 (15.6)	59.6 (15.2)	0.620 (−0.10)
E/A	22	1.2 (0.4)	1.1 (0.4)	0.080 (−0.39)
Peak e′ (lat), cm/s	17	4.8 (0.5)	4.1 (0.5)	<0.001 (−1.39)
Peak a′ (lat), cm/s	15	8.4 (1.2)	6.8 (0.8)	<0.001 (−1.66)
Peak e′ (sep), cm/s	21	5.1 (0.3)	5.0 (0.6)	0.920 (0.08)
E/e′ (lat)	17	14.6 (3.7)	14.9 (3.9)	0.578 (0.08)
E/e′ (sep)	21	14.1 (3.3)	11.64 (2.9)	0.008 (0.77)
Systolic‐diastolic coupling				
S′/e′ (lat)	17	2.86 (1.01)	3.28 (0.92)	0.024 (0.43)
S′/e′ (sep)	21	2.94 (0.94)	2.57 (0.59)	0.158 (−0.46)

*Note*: Data presented as mean (SD). Missing data for tissue Doppler imaging‐derived metrics, including peak S′ (lat, *n* = 4, sep, *n* = 1), peak E′ (lat, *n* = 5, sep, *n* = 1), were due to technical difficulties during data acquisition.

Abbreviations: EDV, end‐diastolic volume; EF, ejection fraction; ESV, end‐systolic volume; *g*, Hedge's effect size.

#### 3.2.2 Hemodynamics

Reductions in aortic SBP (∆ = −9 mmHg, 95% CI: −14 to 4 mmHg, *p* = 0.001) and ESP (∆ = −8 mmHg, 95% CI: −13 to 4 mmHg, *p* < 0.001) were observed post‐firefighting. Brachial pressures were also reduced, but central PWV and AIx75 were unchanged by firefighting (Table 3). Firefighting activity increased HR, reaching a mean peak of 172 (12) b.min^−1^during the firefighting evolution (*p* < 0.001). Elevations in HR were still observed 10 min post‐firefighting compared to baseline (∆ = 21 b.min^−1^, 95% CI: 18–25 b.min^−1^, *p* < 0.001).

**TABLE 3:** Hemodynamic responses to acute live‐firefighting.

	*n*	Pre	Post	*p* (g)
HR, bpm	22	65 (8)	86 (11)	<0.001 (2.25)
ED, ms	20	328 (17)	300 (8)	<0.001 (−1.32)
TR, ms	18	163 (20)	152 (15)	0.021 (−0.57)
CO, L/min	22	5.8 (1.4)	6.3 (1.8)	0.282 (0.31)
bSBP, mmHg	22	132 (14)	123 (13)	0.008 (−0.67)
bDBP, mmHg	22	81 (8)	76 (6)	0.006 (−0.63)
bMAP, mmHg	22	98 (9)	92 (7)	0.002 (−0.72)
bPP, mmHg	22	51 (11)	46 (10)	0.105 (−0.46)
aSBP, mmHg	20	115 (11)	106 (9)	0.001 (−0.89)
ESP, mmHg	20	104 (10)	95 (8)	<0.001 (−0.95)
AP, mmHg	19	3 (3)	1 (2)	0.023 (−0.53)
AIx75, %	19	1 (11)	3 (10)	0.128 (0.23)
Ew ≥0, dyne.s.cm^−2^	11	1730 (785)	633 (485)	<0.001 (−1.35)
TPR, mmHg.L.min^−1^	22	16.91 (5.89)	15.47 (4.30)	0.163 (0.27)
SAC, mL/mmHg	17	2.47 (0.73)	2.36 (0.89)	0.276 (−0.13)
Tau	19	2.52 (0.66)	2.00 (0.48)	<0.001 (−0.87)
cPWV, m/s	20	7.66 (1.21)	7.23 (2.15)	0.403 (−0.24)
SPTI, mmHg × ms	20	43,032 (3569)	36,668 (4264)	<0.001 (−1.59)
DPTI, mmHg × ms	20	49,243 (9973)	31,499 (5497)	<0.001 (−2.31)
Buckberg index	20	1.15 (0.23)	0.87 (0.14)	<0.001 (−1.40)

*Note*: Data presented as mean (SD). *g*: Hedge's effect size. Missing data in tonometry‐derived metric, including TR (*n* = 4), aSBP (*n* = 2), AP (*n* = 3), AIx75 (*n* = 3), SPTI (*n* = 2), DPTI (*n* = 2), and the Buckberg index (*n* = 2), resulted from technical difficulties during acquisition or issues with pulse wave analysis estimation. Missing data for SAC (*n* = 5) and Tau (*n* = 3), parameters modeled from the aortic reservoir function, were due to estimation errors.

Abbreviations: AIx75, augmentation index normalized for 75 bpm; aSBP, aortic systolic blood pressure; AP, augmentation pressure; bDBP, brachial diastolic blood pressure; bMAP, brachial mean arterial pressure; bPP, brachial pulse pressure; bSBP, brachial systolic blood pressure; cPWV, central pulse wave velocity; ED, ejection duration; ESP, end‐systolic pressure; Ew ≥0, wasted pressure effort with values equal to or greater than zero; HR, heart rate; TPR, total peripheral resistance; TR, systolic time of the propagated wave; SAC, systemic arterial compliance.

#### 3.2.3 Ventricular‐arterial coupling, pulsatile afterload, and coronary perfusion

**FIGURE 1:**
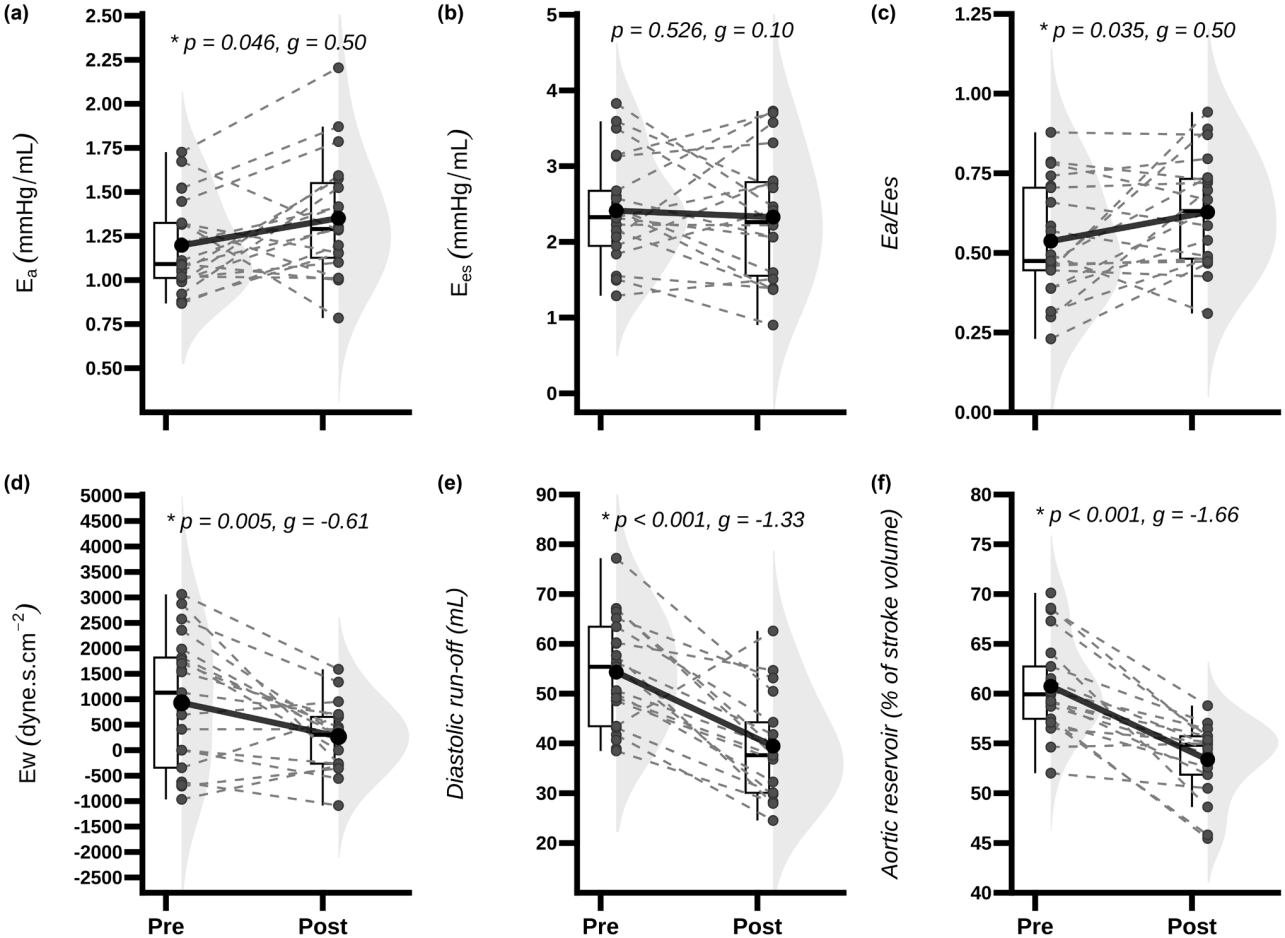
Firefighting‐induced changes of older firefighters in arterial elastance (Ea, a), ventricular elastance (Ees, b), ventricular‐arterial coupling ratio (Ea/Ees, c), wasted pressure effort (Ew, d), diastolic run‐off (e) and aortic reservoir function (f).

The coupling ratio increased after firefighting (∆ = 0.10, 95% CI: 0.01–0.19, *p* = 0.035), resulting from an elevation in Ea (∆ = 0.16 mmHg/mL, 95% CI: 0.01– 0.32, *p* = 0.046), while Ees remained unchanged (Figure 1). Following firefighting, reductions were noted in both Ew (∆ = −800 dyne cm^−2^ s, 95% CI: −1333 to −275, *p* = 0.005) and aortic reservoir function (∆ = −6.9%, 95% CI: −9.2% to −4.5%, *p* < 0.001). Ew analyses, using only positive values, also supported reductions after firefighting (Table 3). Coronary perfusion, as indexed by the Buckberg index (∆ = −0.28, 95% CI: −0.38 to −0.19, *p* < 0.001), was reduced post‐firefighting due to greater reductions in DPTI than SPTI (Table 3). Post‐firefighting decreases in SV were associated with those of diastolic run‐off but (*r* = 0.94, *p* < 0.001) not with Ew (*r* = −0.27, *p* = 0.329). Changes in aortic reservoir function (*r* = 0.92, *p* < 0.001) but not in Ew (*r* = 0.31, *p* = 0.260) were associated with the Buckberg index. Changes in HR were inversely associated with both pulsatile afterload parameters (Ew: *r* = −0.60, *p* = 0.017; and aortic reservoir function: *r* = −0.80, *p* < 0.001) and the Buckberg index (*r* = −0.66, *p* = 0.008). ANCOVA models showed that adjusting for HR eliminated the time effects on Ea (*p* = 0.061), Ew (*p* = 0.572), aortic reservoir function (*p* = 0.616), and the Buckberg index (*p* = 0.506), whereas controlling for hypertension or obesity did not (all time effects *p* < 0.01).

## 4 DISCUSSION

The main findings of this study were that: (1) the ventricular‐arterial coupling ratio increased due to a rise in Ea with no change in Ees, and (2) pulsatile afterload indexed by Ew was reduced, but increased indexed to the aortic reservoir function. As expected, upon completion of firefighting activities, changes in diastolic run‐off were associated with reductions observed in SV, but those of Ew were not. Collectively, our findings suggest that a diminished Windkessel effect, rather than the early wave reflection component of pulsatile afterload, contributes to this post‐firefighting cardiovascular strain and the reduced coronary perfusion noted in middle‐aged firefighters. Still, the role of pulsatile afterload in cardiovascular strain after simulated firefighting activities remains unclear, as observed changes are associated with HR. Indeed, elevations in HR shift reflected waves into diastole, yielding a positive effect on Ew but simultaneously reducing diastolic time, negatively impacting aortic reservoir function and coronary perfusion.

During physical exertion in young healthy adults, adequate ventricular‐arterial coupling to maintain blood ejection and perfusion pressure manifests via a larger increase in Ees than Ea with the resultant ratio reduction favoring stroke work over energetic efficiency compared to rest (Najjar et al., 2004). However, firefighting‐related exertion evokes the opposite response, an increase in the coupling ratio driven by an elevated Ea (Hibner et al., 2022), which this study extends to middle‐aged firefighters. In comparison to the reported responses by our group in younger colleagues, middle‐aged firefighters appear to have smaller firefighting‐induced increases in Ea (0.15 vs. 0.29 mmHg/mL) and Ea/Ees (0.09 vs. 0.019). This discrepancy is most likely due to differences in simulated firefighting stimulus, as younger firefighters completed a 3‐h bout, while middle‐aged firefighters, in this study, did only 18 min. The reported coupling responses in middle‐aged firefighters should not be considered evidence of an abnormal coupling mismatch. Post‐firefighting Ea and Ea/Ees remained within the normative ranges (e.g., Ea/Ees ~ 0.5–1.0), reinforcing the notion that only severe changes in these parameters have prognostic value, as observed in systolic dysfunction (Chirinos, 2013; De Tombe et al., 1993; Ikonomidis et al., 2019). Controlling for post‐firefighting elevations of HR abolished increases in Ea, further supporting its dependence on HR, and that Ea is not a true index of general arterial load (Chemla et al., 2003; Segers et al., 2002). Indeed, the nonsignificant TPR reduction linked to the post‐firefighting hypotensive response suggests that resistive afterload was not increased.

The present study is the first to document the post‐firefighting response of pulsatile afterload considering both wave reflection (i.e., Ew) and the Windkessel effect (i.e., aortic reservoir function). Contrary to our hypothesis, the observed decrease in Ew indicates that aortic pressure augmentation from wave reflection in systole does not contribute to the reduced SV and cardiac strain after live firefighting. Such a conclusion is further substantiated by the small reduction in augmentation pressure and unchanged AIx post‐firefighting. Although our middle‐aged firefighters exhibited the expected age‐related arterial stiffening with possibly faster‐reflected waves returning to the ascending aorta, firefighting did not change cPWV, aligning with some (Fahs et al., 2011) but not all reports in younger firefighters (Fernhall et al., 2012; Hibner et al., 2022). The post‐firefighting reduction in Ew mirrors the wave reflection response to exercise‐induced heat stress (Lefferts et al., 2015), likely driven by an elevated HR resulting in shorter systolic ejection and wave propagation time. Indeed, firefighting‐induced changes in HR were inversely related to those of Ew, reinforcing that elevated HR causes a temporal shift with reflected waves arriving in diastole (Wilkinson et al., 2000). Importantly, the impact of discrete wave reflection compared to aortic reservoir function on pulsatile afterload appears less than once thought because of wave dispersion along the aorta and peripheral entrapment of reflected waves (Schultz et al., 2013, 2014; Sharman et al., 2009). The aortic reservoir function is suggested as the main determinant of pulsatile afterload (Davies et al., 2010; Schultz et al., 2014), and after firefighting, it was diminished due to reduced diastolic run‐off and SV. While the post‐firefighting elevation in HR leading to a shortening in diastolic time was associated with a reduced aortic reservoir function, it is also conceivable that a dehydration‐induced fall in preload and, thus, SV contributes to depressed diastolic run‐off (Heffernan et al., 2010). In contrast, systemic arterial compliance—the key determinant of aortic reservoir function—remained unchanged after firefighting, similar to responses observed following aerobic exercise (Lane et al., 2012). Taken together, the pressure buffering capacity of the aorta appears preserved, but due to the post‐firefighting hypotensive and positive chronotropic response, the aortic inflow was reduced, underlying reductions in coronary perfusion.

Although post‐firefighting HR was a statistical confounder of pulsatile afterload responses, its physiological importance should not be disregarded, as elevated HR contributes to the cardiovascular strain of firefighting (Kales & Smith, 2017; Smith et al., 2001). The elevated HR observed post‐firefighting reduces ventricular filling time and may contribute to the reduction in SV. These observations indicate that cardiovascular drift persists shortly after completing live‐fire firefighting activities, which could be aggravated considering the potential for hyperthermia‐induced dehydration (Gonza et al., 2000; Gonzalez‐Alonso et al., 1995). However, in our study, the link between HR and SV is refuted as firefighting‐induced elevations in HR were not associated with EDV (*r* = 0.29, *p* = 0.300) or SV (*r* = 0.37, *p* = 0.169) changes, aligning with evidence derived from prolonged endurance exercise. Our group has shown that prolonged exposure to repeated bouts of firefighting activities (3 h) produces consistent changes with exercise‐induced cardiac fatigue, with a reduction in diastolic rather than systolic function contributing more to post‐firefighting reductions in SV (Fernhall et al., 2012). The present study expands such a notion to shorter‐duration firefighting activities in middle‐aged firefighters, suggesting that effort intensity has an important influence on cardiac fatigue, agreeing with recent exercise reports (Coates et al., 2023). Despite cardiac fatigue being mechanistically linked to oxidative stress‐induced cardiomyocyte damage and sympathetic overactivity (Hart et al., 2006; Vitiello et al., 2011), it is plausible that deficits in systolic‐diastolic coupling could also play a role.

Systolic‐diastolic coupling reflects how energy deposition from systolic contraction primes early diastolic re‐lengthening and suction, independent of atrioventricular pressure gradients (Friedberg et al., 2016; MacNamara et al., 2021; Nikolic et al., 1988). After firefighting activities, systolic contraction (i.e., peak S′) was unchanged, but lateral early diastolic re‐lengthening (i.e., peak e′) was reduced, leading to altered systolic‐diastolic coupling (i.e., increased S′/e′). These observations suggest that the heart is unable to reuse systolic energy for diastolic elastic recoil and early filling in a region‐specific manner, despite elevated septal and lateral S′ values (>10 cm/s) (Chahal et al., 2010), a likely compensatory response to diastolic dysfunction. Despite TDI markers being less load‐dependent (MacNamara et al., 2021) and supporting systolic‐diastolic coupling as a plausible key component of cardiovascular strain, the firefighting‐induced reduction in preload likely contributes to cardiac changes observed. The post‐firefighting reduction in EDV indirectly suggests reductions in preload, reinforcing that the decrease in SV is primarily mediated by the Frank‐Starling mechanism, given the unaltered contractility (i.e., Ees and peak S′) and reduced afterload (i.e., MAP).

### 4.1 Implications

The role of ventricular‐arterial interactions in the increased risk of sudden cardiac events after firefighting is unclear. While we observed reductions in coronary perfusion that could trigger arrhythmias and angina (Bolli et al., 1986; Fox & Ferrari, 2011), such ischemic changes are linked to elevated HR rather than firefighting‐induced changes in pulsatile (i.e., Ew) and general (i.e., Ea, MAP) afterload (Lefferts et al., 2015). Delayed HR recovery is a key predictor of cardiac events and mortality (Qiu et al., 2017), emphasizing the need for post‐firefighting monitoring. Upon completion of firefighting activities, diastolic dysfunction was exacerbated, which is shown to have prognostic value for arrhythmia based on evidence of physical exertion in older adults (Takagi et al., 2012). Although middle‐aged firefighters are expected to experience greater cardiovascular strain due to higher cardiometabolic risk, indirect comparisons with our work in younger colleagues suggest similar post‐firefighting drill responses in ventricular‐arterial coupling, and cardiac function.

### 4.2 Experimental considerations and limitations

We estimated left ventricular volumes using the parasternal long‐axis view, even though Simpson's disc method from a 4‐chamber view is considered optimal. M‐mode, however, provides reliable estimates and is used to assess cardiac volume responses to simulated firefighting activities (Fernhall et al., 2012; Hibner et al., 2022). Pulsatile afterload is preferably quantified by integrating pressure‐flow relationships rather than by pressure‐alone wave separation algorithms (Shenouda et al., 2021). In fact, pressure‐only algorithms can yield physiologically implausible negative Ew, yet analyses limited to positive values supported firefighting‐induced reductions. Of note, assessments in the supine position confound the post‐firefighting cardiovascular response. In particular, the reported reductions in SV may be more pronounced in seated recovery due to venous pooling (Frey et al., 1994), and the elevated HR is expected to be greater. Still, we believe our findings accurately capture the direction of post‐firefighting‐induced changes, but acknowledge that their magnitude is position‐dependent and calls for standardization efforts. Another key limitation is the absence of a comparator arm, which prevents determining the independent effects of firefighting exercise drills versus those of protective gear/clothing on cardiovascular strain. While live, realistic firefighting scenarios maximize ecological validity, they fail to identify potential targets to attenuate such strain—an important area for future research.

The lack of a young group limits insights into whether aging amplifies the cardiovascular strain of firefighting. Additionally, studying only middle‐aged male firefighters restricts the generalizability of findings to females. Lastly, the small sample size and missing data limit statistical power, particularly for covariate control. Indeed, hypertension, obesity, and diastolic dysfunction were prevalent among our middle‐aged firefighters, known to impact ventricular‐arterial coupling and pulsatile afterload. Although controlling for baseline effects did not change responses to firefighting activities, it remains unclear whether the firefighting‐induced strain is due to environmental stressors, underlying cardiovascular risk factors in middle‐aged firefighters, or a combination of both.

## 5 CONCLUSIONS

Although middle‐aged firefighters exhibited the typical cardiovascular strain upon completing firefighting activities, including reduced diastolic function and coronary perfusion, the role of ventricular‐arterial interactions and pulsatile afterload on these responses remains unclear. The elevated HR after firefighting drills confounded the small increase in ventricular‐arterial coupling ratio due to increased Ea, along with reduced Ew and aortic reservoir function. Future research with large samples is needed to clarify the mechanisms underlying heightened cardiovascular risk after firefighting.

## AUTHOR CONTRIBUTIONS

J.L.M. drafted the manuscript; B.F., G.P.H, and D.S. conceived and designed research; A.D.L., S.M.R., H.Y., and K.B. performed experiments; J.L.M. analyzed data; J.L.M., T.B., and B.F. interpreted results of experiments; J.L.M. prepared figures; A.D.L., S.M.R., H.Y., K.B., G. P. H., D.S., T.B., and B.F. edited and revised the manuscript; all authors approved the final version of the manuscript.

## FUNDING INFORMATION

This study was funded by the Department of Homeland Security: EMW‐2009‐FP‐00544, awarded to G.P.H.

## CONFLICT OF INTEREST STATEMENT

The authors declare no conflicts of interest.

## ETHICS STATEMENT

The study was approved by the IRB and conformed to the Declaration of Helsinki and all particiapnts signed informed consent.

## Data Availability

The data that support the findings of this study are available from the corresponding author, BF, upon reasonable request.
